# Descriptive Analysis of Heavy Metals Content of Beef From Eastern Uganda and Their Safety for Public Consumption

**DOI:** 10.3389/fnut.2021.592340

**Published:** 2021-02-11

**Authors:** Keneth Iceland Kasozi, Yunusu Hamira, Gerald Zirintunda, Khalaf F. Alsharif, Farag M. A. Altalbawy, Justine Ekou, Andrew Tamale, Kevin Matama, Fred Ssempijja, Robert Muyinda, Francis Kawooya, Theophilus Pius, Hellen Kisakye, Paul Bogere, Henry Matovu, Leonard Omadang, Patrick Etiang, Joseph Mbogua, Juma John Ochieng, Lawrence Obado Osuwat, Regan Mujinya, Gaber El-Saber Batiha, Ochan Otim

**Affiliations:** ^1^Department of Animal Production and Management, Faculty of Agriculture and Animal Sciences, Busitema University Arapai Campus, Soroti, Uganda; ^2^School of Medicine, Kabale University, Kabale, Uganda; ^3^Department of Clinical Laboratory Sciences, College of Applied Medical Sciences, Taif University, Taif, Saudi Arabia; ^4^National Institute of Laser Enhanced Sciences, Cairo University, Giza, Egypt; ^5^Department of Wildlife and Aquatic Animal Resources, School of Veterinary Medicine and Animal Resources, College of Veterinary Medicine, Animal Resources and Biosecurity, Makerere University, Kampala, Uganda; ^6^School of Pharmacy, Kampala International University Western Campus, Bushenyi, Uganda; ^7^Faculty of Biomedical Sciences, Kampala International University Western Campus, Bushenyi, Uganda; ^8^Department of Medical Laboratory Sciences, School of Allied Health Sciences, Kampala International University Western Campus, Bushenyi, Uganda; ^9^Faculty of Agriculture and Environmental Science, Muni University, Arua, Uganda; ^10^School of Medicine and Health Sciences, Soroti University, Soroti, Uganda; ^11^Department of Pharmacology and Therapeutics, Faculty of Veterinary Medicine, Damanhour University, Damanhour, Egypt; ^12^Department of Chemistry, Faculty of Science, Gulu University, Gulu, Uganda; ^13^Department of Humanities and Sciences, University of California, Los Angeles, Los Angeles, CA, United States

**Keywords:** heavy metals, food hygiene, food safety, Uganda, beef industry

## Abstract

In this study, we initiated an effort to generate information about beef safety in Uganda. Our entry point was to assess by atomic absorption spectrophotometry the levels of essential elements copper (Cu), cobalt (Co), iron (Fe) and zinc (Zn), and non-essential elements lead (Pb), chromium (Cr), nickel (Ni), and cadmium (Cd) in 40 beef samples collected from within and around Soroti (Uganda). The information was used to evaluate the safety of consuming such beef against the World Health Organization (WHO) limits. The latter was accomplished by (i) estimating the daily intake (EDI) of each metal in the study area, (ii) modeling the non-cancer health risk using the target hazard quotient (THQ) and (iii) modeling the cancer risk using the incremental lifetime cancer risk (ILCR). The study finds that the mean concentrations (±95% CI) and EDI were in the order of Fe > Zn > Cr > Ni > Pb > Co > Cu > Cd. Cancer risk was found to be due to Ni > Cr > Cd > Pb and significantly higher in children than adults. The latter particularly demonstrates the importance of Ni poisoning in the study area. Overall, while essential elements in our beef samples were below WHO limits (hence no health risks), non-essential elements had high health and cancer risks due to higher levels of Cr and Ni.

## Introduction

Contamination of food by heavy metals such as lead (Pb), iron (Fe), chromium (Cr), cadmium (Cd), copper (Cu), cobalt (Co), Nickel (Ni) and zinc (Zn) in developing countries is a major public health problem (1, 2). This has been demonstrated in Uganda, for example, in beef (3, 4), in drinking water (5, 6), street food (7), alcoholic beverages (8, 9), fish (10), food crops grown at dumpsites (11), and in food consumed around Lake Victoria and Lake George (12–14). Compounding this is human activities such as mining, irrational usage of chemicals and poor policies on industrial waste management which only exacerbate the problem (15, 16). In Africa, there is a general absence of legislations clearly designed to mitigate environmental degradation. This only suggests one natural course for the continent: that environmental contamination with heavy metals will worsen.

In the body, essential elements in moderate amounts play a crucial role in cellular functions. Their bioavailability is required for certain enzymes to aid metabolic reactions in cellular systems. Iron for example, once complexed with Cu, helps in electron transport system during ATP metabolism [see Supplementary Information in (17)]. The World Health Organization (WHO) recommends a daily intake of 6 ppm/day for Fe and 1 ppm for Cu in children. For a 70 kg adult, the recommendation is 392 ppm/week for Fe and 245 ppm/week for Cu (18). Excessive human consumption of Cu can disrupt physiological processes in the digestive and excretory systems leading to intestinal ulcerations and tubular necrosis (19, 20). Concentrations of Fe and Cu in beef samples are 184 and 160 ppm, respectively (21). Furthermore, contamination of red meat with heavy metals has been identified as a major public health risk requiring novel strategies to decontaminate meat (22).

Zn, an importance micronutrient, is an essential co-factor in living cells. It is required in maintaining the integrity of cellular structure, and as a regulator of cellular activities associated with strong antioxidant capacity (23, 24). The recommended maximum consumption of Zn is 7 ppm/week for a child (18) and 490 ppm/week for a 70 kg adult (25). The concentration of Zn in beef has been shown to be 127 ppm (21). This level of Zn is excessive when absorbed into the body since Zn suppresses Cu and Fe absorption leading to deficiencies of these elements in cells. As a consequence, ingestion of high Zn concentrations in a diet leads to gastrointestinal disorders and dysregulation of lipid metabolism in the body (26). Co in the form of vitamin B_12_ (26), is usually present at 0.25 ppm on in beef average (21) and acts as an essential nutrient for managing anemia associated with malabsorption of vitamin B_12_ in humans during ulcers (27). Co toxicity mainly leads to cardiovascular, hematological, neurological, and endocrine deficits. (28, 29).

Non-essential elements such as Pb, Cd, Ni and Cr are toxic to the body. Pb exposure for example is associated with gastrointestinal irritation and neurotoxicity in children and adults while chronic exposure may lead to cancer in humans (30, 31). These metals, Cr and Ni for example, arise from tanneries and from black smith industry, respectively, in Uganda. WHO recommends a tolerable intake of 0.025 ppm/week for Pb (32), a change from a previous permissible limit of 1.75 ppm/week for a 70 kg adult (18). Cd is a carcinogenic agent known to induce some form of cancer (33). This includes those of the lung and the prostate. WHO has set a permissible limit of 0.49 ppm/body weight/week for Cd (21, 34). In 2003, the Food and Agricultural Organization (FAO) set a weekly tolerable intake of 7 ppm/body weight. The European Food Safety Agency (EFSA) modified this to 2.5 ppm/body weight/week (21), and then to 0.49 ppm/bodyweight/week day (34) as a more practical limit. Ni is important in the body at low levels as an enzyme activator, but a carcinogen at high concentration (35, 36). WHO has set a Ni tolerable daily intake of 11 ppm/day/body weight for humans (18). Cr has been associated with urticaria, anemia and generalized visceral disorders (37, 38). In a recent study, half of a local population which consumed meat of wild boar was shown to be exposed to levels of Cr >12.5l ppm/week/ person (CI for the median = 0.5l ppm/Cr week/person) (39), much lower than the intake of 31.67 and 28.47 ppm/Cr week/person in both men and women in Nigeria (40). The latter demonstrates the importance of Cr in food safety.

Heavy metals enter the meat though feedlots and water used in raising the donor animals (3, 41). Furthermore, environmental pollution with sewage and urban agriculture which thrives on it helps propagate heavy metal bio-accumulation in foods (11, 42).

In developing countries, information concerning the extent of heavy metal contamination is scarce. Policy makers in the food industry are therefore not well informed to take the necessary steps to protect consumers. There is therefore need to start providing this critical of information for safeguarding public safety. In this study, we begin the process filling this information gap by establishing the heavy metal concentrations in beef consumed in a typical developing country community. We further show how to assess public health risk posed by consuming contaminants at the levels detected in beef.

## Methods

### Study Design

This study was an observational one conducted in the Soroti district of eastern Uganda during the months of December 2019 to March 2020. The area was chosen because of its cattle keeping culture, along with the capacity to study agriculturally-related problems in a purposely located university in Soroti. Furthermore, Soroti district has numerous butcheries (points of sale) to acquire samples. The belief in the area, anecdotally at best, is that beef sold there are contaminated with heavy metals (among others), the sources of which are anthropogenic in nature.

A total of 40 beef samples were collected from butcher points of sale in local villages. The georeferenced coordinates for each village with an accuracy <3 m were also taken (data files available). These were used for mapping (quantum geographical systems, qGIS®, version 3.03 Cirona) onto images acquired using the Sentinel-2A satellite from the United States Geographical Surveys (Sentinel-2 image ID: L1C_T36NWG_A023913_20200120T080825 and image ID: L1C_T36NWH_AO23484_20191221T080920). The following modifications were made in the process: Band 1 was set to red, interpolation was set to linear, color ramp was set to viridis. These settings were divided to show 6 categories: black (26 units) for deep water = Lake Kyoga, purple (54 units) for shallow water =swamps, light blue (82 units) for moderate vegetation, green (110 units) for sparse vegetation and yellow (138 units) for bare/dry soil in qGIS®.

### Preparation of Heavy Metal Standards

Solutions of heavy metals working standards were prepared from stock solutions as previously described (3). For instrument calibration, four (4) calibration standard solutions were prepared at the 0.0, 0.5, 2.0, and 5.0 ppm levels to cover the linear absorbance range of the atomic absorption spectrophotometer used in this study (AAS, PerkinElmer 2380; see Table 1 for outcome).

**Table 1:** The wavelengths and corresponding slit widths used to obtain instrumental linear calibration range for each metal.

**Metal**	**λ, nm**	**Slit width, nm**	**Slope**	**Intercept**	***R*^**2**^**
Pb	217.0	1.0	0.0168	0.0082	0.976
Cr	357.9	0.2	0.0193	0.0067	0.979
Cu	324.9	0.5	0.1152	0.0034	1.000
Zn	213.9	1.0	0.2051	0.1166	0.921
Cd	228.8	0.5	0.2075	0.0884	0.956
Co	240.7	0.3	0.0332	0.0110	0.984
Fe	248.3	0.2	0.0304	0.0112	0.982
Ni	323.0	0.2	0.0362	0.0125	0.984

*The slopes and the intercepts extracted from absorbance vs. concentration scatter plots (y = mx + c; y = absorbance, m = slope, x = concentration and c = intercept on y axis when x = 0), and the corresponding coefficients of determination (R^2^) are also given*.

### Sampling, Preparation, and Analysis of Beef

About 200 g of raw beef samples were collected from the butchers during morning slaughter hours and placed into sterile plastic bottles. These were transported at 4°C to our laboratory for processing. Analysis of the processed samples was based on standard methods for heavy metal determination (3, 43). The concentration of the heavy metals in each beef extract was determined using AAS at wavelength corresponding to, and instrumental linear calibration range for, each metal listed in Table 1. Blank (solvent) analyses were done as well to assure laboratory control over the analytical process. This was inline with standard practice in this field (44).

### Modeling of Estimated Daily Intake of Heavy Metals in Beef

To estimate the daily intake of heavy metals from consuming beef from Soroti on the basis of samples collected, the following equation was used;

(1) $$\begin{array}{r} EDI=\frac{C\times IR}{BW} \end{array}$$

where, EDI = estimated daily intake, C = heavy metal concentration and BW = body weight (60.7 and 20.5 kg for adults and children, respectively). The beef ingestion rate (IR) for Ugandans is 120 and 56.7 g for both adults and children, respectively (3).

### Modeling Non-cancer Risk Associated With Beef Consumption

The target hazard quotient (THQ) was used to generate the hazard index (HI) to determine presence of non-carcinogenic health effects following oral ingestion of beef using the following equation:

(2) $$\begin{array}{r} THQ=\frac{CDI}{RfD}, \end{array}$$

where CDI is the chronic daily intake obtained using the equation below, and RfD is the oral reference dose of the contaminant, an estimation of the maximum permissible risk on human population through daily exposure.

(3) $$\begin{array}{r} CDI=\frac{EDI\;\times \;EFr\;\times \;EDtot\;}{AT}, \end{array}$$

where EDI is the estimated daily intake of a metal via ingestion of specific route. EFr is the exposure frequency (365 days/year); EDtot is the exposure duration (i.e., 6 years for children and 30 years for adults); and AT is the period of exposure for noncarcinogenic effects. For non-cancer risk modeling, AT = EFr × EDtot (2,190 days in children and 10,950 days in adults) and the reference dose (RfD) for each hazard was described previously (5). i.e., 0.04, 0.3, 0.0035, 0.0005, 0.03, 0.7, 0.02, and 0.0003 ppm/day for Cu, Zn, Pb, Cd, Co, Fe, Ni, and Cr, respectively. Exposure to multiple contaminants results in additive and interactive effects; thus, the hazard index (HI = ∑THQ) was used as an indication of risk (5).

### Modeling Cancer Risk Associated With Beef Consumption

The incremental lifetime cancer risk (ILCR) using standard methods (3) was used to model the cancer risk in the Ugandan population using the following equation:

(4) $$\begin{array}{r} ILCR=CDI\times CSF, \end{array}$$

where CDI is the chronic daily intake of a particular metal and this was estimated over the 70-year lifespan for Ugandans (i.e., AT =70 years × 365 days = 25,550 days). In addition, the cancer slope factor (CSF) of 0.0085 (ppm/day)^−1^, 0.38 (ppm/day)^−1^, 0.84 (ppm/day)^−1^, and 0.5 (ppm/day)^−1^, for Pb, Cd, Ni, and Cr respectively, were used (45, 46).

### Statistical Analysis

Descriptive statistical analyses were conducted in MS Excel version 2010. A one sample *t*-test was conducted by making comparisons using the WHO/FAO reference values and significance was reported when *P* < 0.05. A summary was acquired by getting the discrepancy between the actual and theoretical mean (WHO limits) values to define “High” and “Low.” On the modeled estimates, a two sample *t*-test was conducted between children and adults on the estimated daily intake and cancer risk. Furthermore, presence of cancer risk was identified when thee ILCR was >1 × 10^−4^. Superscript “a” was used to indicate absence of risk while superscript “b” was used to indicate the presence of risk in the sampled population. Hierarchical clustering on principal components (HCPC) (47) was used to assess natural clustering of data. HCPC combines the benefits of both principal components analysis (PCA) with those of cluster analysis (CA) to group individuals or variables. Factoshiny package running within RStudio platform was used for HCPC (48, 49).

## Results

### Description of The Study Area

**Figure 1:**
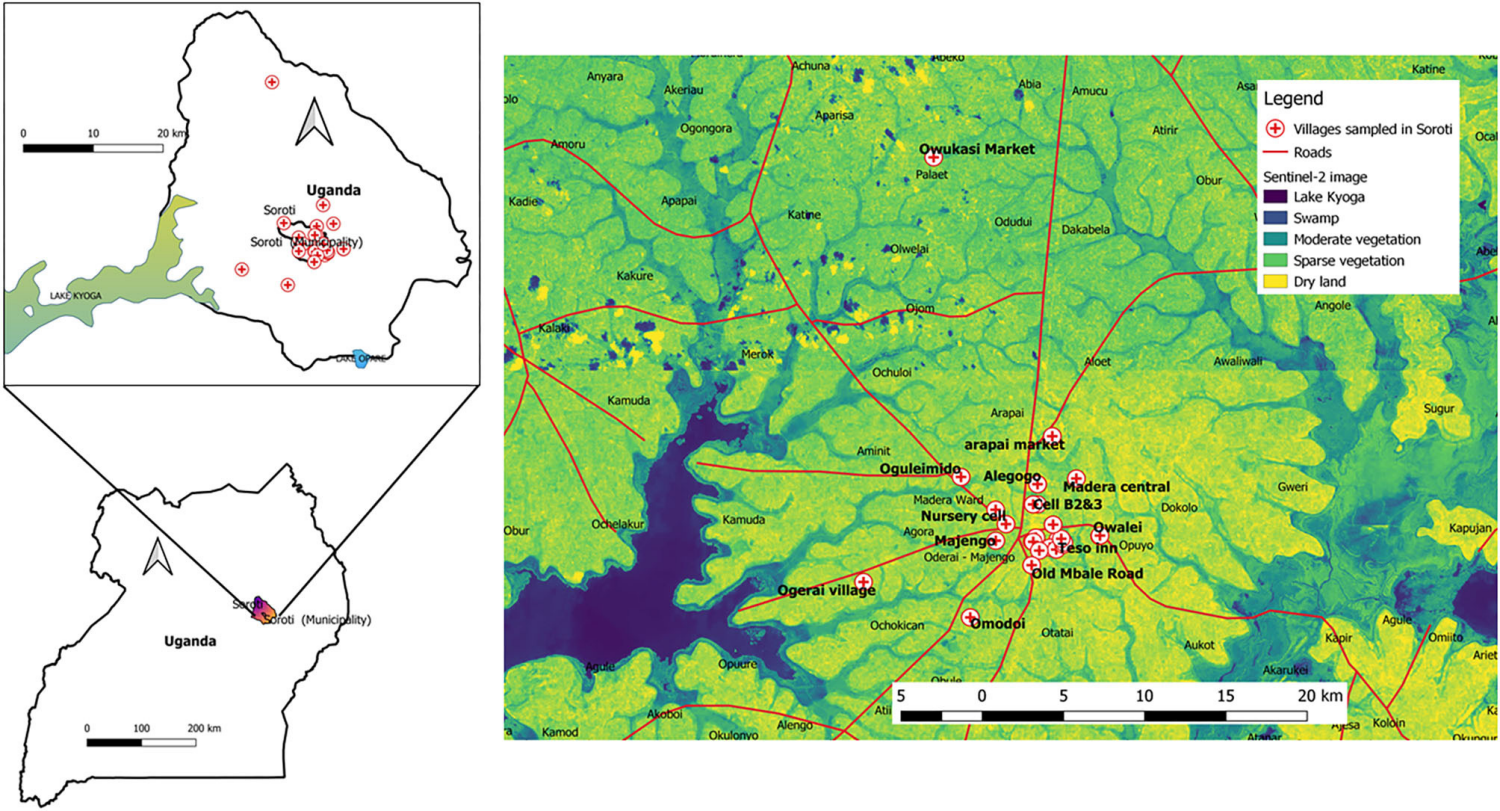
Map of Uganda showing vegetation distribution in the study area. Soroti is the largest district in the Teso sub-region and it is ~116km from Mbale, the largest city in Eastern Uganda. Soroti has a population of about 114,000 and this agricultural area is known for its high productivity of millet, cassava, peas, potatoes, beans, onions, tomatoes, cabbages and simsim as a result of its close proximity to the Lake Kyoga basin. Soroti also lies within the cattle corridor of Uganda where indigenous zebu cattle constitute the major animals kept in rural homesteads.

The study was conducted in an agricultural area with a high distribution of sparse green vegetation and water tributaries from Lake Kyoga. These conditions are conducive to livestock productivity in areas shown in Figure 1.

### Description of Heavy Metal Concentrations in Beef From Eastern Uganda

The statistics associated with metals concentrations in this study are displayed in Table 2. It can be seen from the table that only Cd level overall was below the WHO guideline maximum value and, as such, safe in a beef serving in the study area.

**Table 2:** Attributes of heavy metal concentrations in beef of Soroti.

**Variables (ppm)**	**Cu**	**Zn**	**Pb**	**Cd**	**Co**	**Fe**	**Ni**	**Cr**
Mean	1.45	44.43	5.42	0.41	1.63	164.33	14.96	19.37
Standard Error	0.10	2.25	0.28	0.03	0.08	35.51	1.00	1.22
Median	1.32	42.34	5.10	0.37	1.59	121.08	14.07	17.52
Standard Deviation	0.64	14.25	1.77	0.19	0.49	224.60	6.31	7.72
Range	2.90	57.78	8.53	0.87	2.20	1,445.38	20.61	28.94
Minimum	0.70	21.47	2.41	0.11	0.84	65.37	5.97	6.94
Maximum	3.60	79.25	10.94	0.98	3.04	1,510.75	26.58	35.89
25% Percentile	0.98	35.52	4.27	0.30	13.00	83.33	8.97	13.00
75% Percentile	1.75	52.32	6.52	0.49	25.67	173.1	20.71	25.67
Count	40	40	40	40	40	40	40	40
Confidence Level (95.0%)	0.20	4.56	0.57	0.06	0.16	71.83	2.02	2.47
WHO/FAO limits	0.5	1.0	0.1	0.5	0.5	0.01	1.0	0.05
One sample *t*-test *p*-value	<0.0001	<0.0001	<0.0001	0.0066	<0.0001	<0.0001	<0.0001	<0.0001
Discrepancy (Safety)	High(Unsafe)	High(Unsafe)	High(Unsafe)	Low(Safe)	High(Unsafe)	High(Unsafe)	High(Unsafe)	High(Unsafe)

### Description on Estimated Daily Intake of Heavy Metals From Beef of Eastern Uganda

Oral ingestion of Cu, Zn, Pb, Cd, Co, Ni, and Cr were significantly higher in children (*P* < 0.05) than adults. The EDIs are shown in Table 3.

**Table 3:** Modeled estimated daily intake of heavy metals in beef amongst adults and children of Uganda.

**Parameters**	**Estimated daily intake of heavy metals (ppm/day) × 10^−2^**							
	**Cu**	**Zn**	**Pb**	**Cd**	**Co**	**Fe**	**Ni**	**Cr**
**Adults**								
Mean	0.29	8.78	1.07	0.08	0.32	32.49	2.96	3.83
Median	0.26	8.37	1.01	0.07	0.32	23.94	2.78	3.46
Minimum	0.14	4.24	0.48	0.02	0.17	12.92	1.18	1.37
Maximum	0.71	15.67	2.16	0.19	0.60	298.67	5.26	7.09
25% Percentile	0.19	7.02	0.84	0.06	0.25	16.47	1.77	2.57
75% Percentile	0.35	10.34	1.29	0.10	0.37	34.22	4.10	5.08
Count	40	40	40	40	40	40	40	40
Confidence Level (95.0%)	0.04	0.90	0.11	0.01	0.03	142.01	0.40	0.49
**Children**								
Mean	0.40	12.29	1.50	0.11	0.45	45.45	4.14	5.36
Median	0.36	11.71	1.41	0.10	0.44	33.49	3.89	4.85
Minimum	0.19	5.94	0.67	0.03	0.23	18.08	1.65	1.92
Maximum	0.10	21.92	3.03	0.27	0.84	417.85	7.35	9.93
25% Percentile	0.27	9.82	1.18	0.08	0.35	23.05	2.48	3.60
75% Percentile	0.48	14.47	1.80	0.14	0.52	47.88	5.73	0.10
Count	40	40	40	40	40	40	40	40
Confidence Level (95.0%)	0.06	1.26	0.16	0.02	0.0043	19.87	0.56	0.68
Two sample *t*-test *p*-value	0.001320	1.967E-05	2.600E-05	0.002204	6.152E-06	0.2866	0.0008616	0.0004508

**Figure 2:**
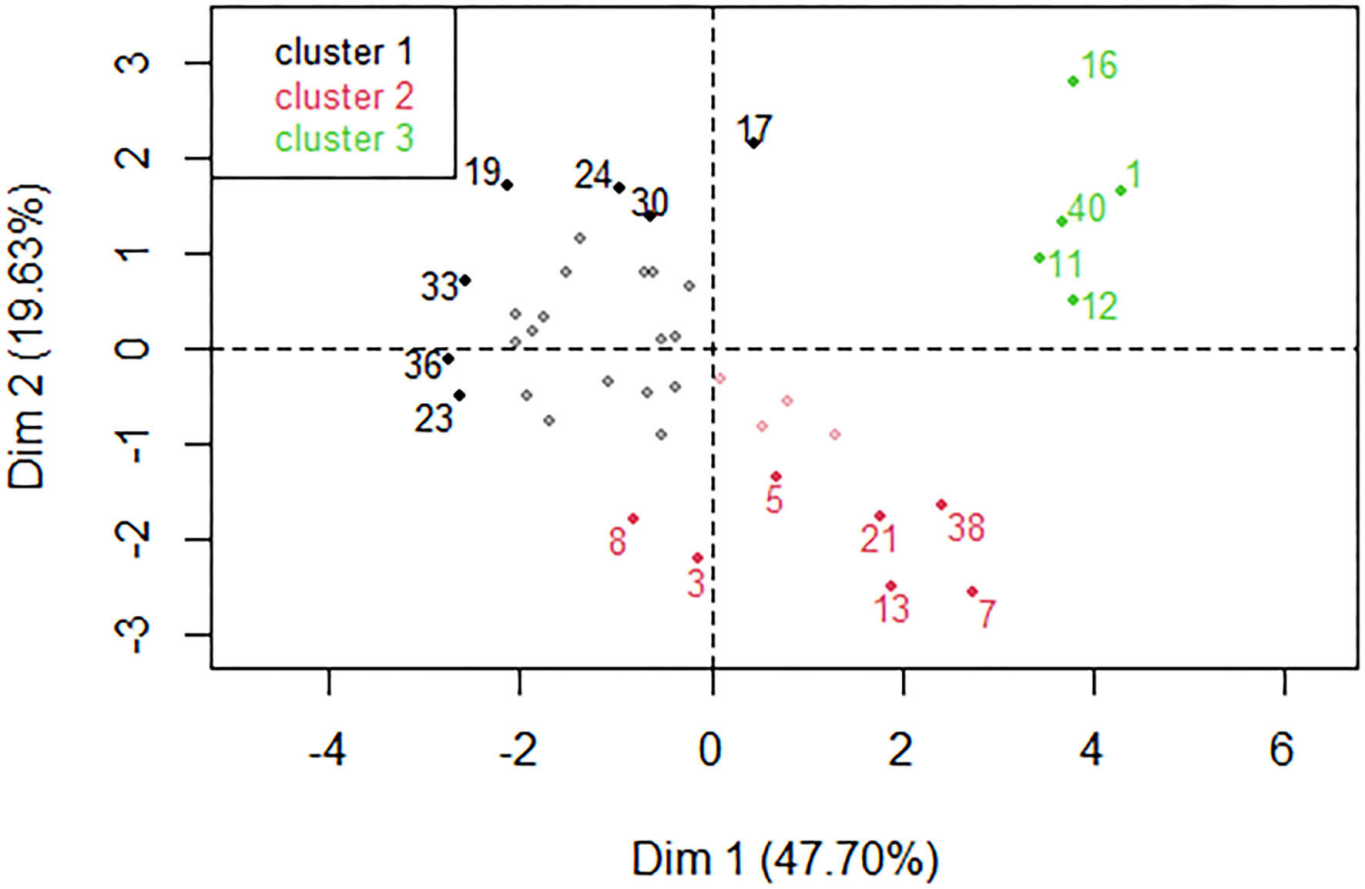
Hierarchical classification of the individual samples by HCPC revealed three clusters color-coded black (cluster 1), red (cluster 2) and green (cluster 3). A few individuals with the strongest influence in each cluster are shown for illustrative purposes.

The estimated daily intake of heavy metals by both children and adults were found to fall into three groups by HCPC (Figure 2). In one EDI group, labeled cluster 1, samples had low levels of Ni, Cr, Cu, and Co. In another (cluster 2), samples had high levels of Cr, Ni, and Cu, but low level of Pb, and in the last group (cluster 3), samples had high levels of Cd, Co, Pb, Ni, Fe, and Cu.

### Modeled Non-cancer Risk Amongst Ugandans

The target hazard quotient (THQ) for the metals studied were above 1 for both children and adults indicating a non-carcinogenic risk. There were no significant differences in THQ values in both adults and children as shown in Table 4.

**Table 4:** Estimation of non-cancer health risks amongst Ugandans associated with oral consumption of beef from Soroti.

**Parameters**	**Target hazard quotient (THQ) × 10^−2^**								**∑ THQ = HI**
	**Cu**	**Zn**	**Pb**	**Cd**	**Co**	**Fe**	**Ni**	**Cr**	
**Adults**									
Mean	7.18	29.28	306.09	163.44	10.72	46.41	147.87	12,766.32	134.77
Median	6.52	27.90	288.17	146.53	10.50	34.20	139.08	11,544.07	121.97
Minimum	3.46	14.15	136.11	42.06	5.56	18.46	59.02	4,574.46	48.53
Maximum	17.79	52.22	617.93	386.79	20.06	426.67	262.76	23,648.16	254.32
25% Percentile	4.87	23.41	241.2	120.1	8.27	23.53	88.68	8,564.00	90.74
75% Percentile	8.63	34.48	368.0	195.6	12.44	48.89	204.70	16,920.00	177.93
Count	40	40	40	40	40	40	40	40	320
Confidence Level (95.0%)	1.01	3.00	31.92	241.4	1.03	20.29	19.94	16.2785	17.29
**Children**									
Mean	10.05	40.97	428.24	228.67	15.00	64.93	206.88	17,860.87	188.56
Median	9.121	39.04	403.16	205.00	14.69	47.84	194.58	16,150.85	170.64
Minimum	4.85	19.79	190.42	58.85	07.78	25.83	82.57	6,399.95	67.90
Maximum	24.89	73.06	864.53	541.14	28.06	596.93	367.61	33,085.21	355.81
25% Percentile	6.81	32.75	337.50	168.10	11.57	32.92	124.1	11,980.00	126.94
75% Percentile	12.07	48.24	514.90	273.60	17.40	68.40	286.4	23,670.00	248.91
Count	40	40	40	40	40	40	40	40	320
Confidence Level (95.0%)	1.41	4.20	44.65	33.78	1.44	28.38	27.89	2,277.46	24.19
Two sample *t*-test	0.0013	<0.0001	<0.0001	0.0021	<0.0001	0.2862	0.0008	0.0004	

### Modeled Cancer Risk Amongst Ugandans

The cancer risk was significantly lowest in the order of Pb < Cd < Cr < Ni in adults than children. Cancer risk following consumption of chromium and Nickel as shown in Table 5.

**Table 5:** Cancer risk associated with oral consumption of beef from Soroti amongst adults and children of Uganda.

**Parameters**	**Incremental lifetime cancer risk (ILCR) × 10^−4^**				**∑ ILCR**
	**Pb**	**Cd**	**Ni**	**Cr**	
**Adults**					
Mean	0.39^a^	17.45^b^	106.50^b^	82.07^b^	206.41^b^
Median	0.37^a^	16.43^b^	100.10^b^	74.21^b^	191.11^b^
Minimum	0.17^a^	7.76^b^	42.49^b^	29.41^b^	79.83^b^
Maximum	0.79^a^	35.22^b^	189.2^b^	152.0^b^	377.21^b^
25% Percentile	0.31^a^	13.75^b^	63.85^b^	55.06^b^	132.97^b^
75% Percentile	0.47^a^	20.98^b^	147.40^b^	108.80^b^	277.65^b^
Count	40	40	40	40	160
Confidence Level (95.0%)	0.04^a^	1.82^b^	14.36^b^	10.47^b^	26.68^b^
**Children**					
Mean	0.11^a^	0.37^a^	29.79^b^	22.96^b^	53.23^b^
Median	0.10^a^	0.33^a^	28.02^b^	20.77^b^	49.23^b^
Minimum	0.05^a^	0.10^a^	11.89^b^	8.229^b^	20.26^b^
Maximum	0.22^a^	0.88^a^	52.94^b^	42.54^b^	96.58^b^
25% Percentile	0.09^a^	0.27^a^	17.87^b^	15.41^b^	33.64^b^
75% Percentile	0.13^a^	0.45^a^	41.25^b^	30.43^b^	72.26^b^
Count	40	40	40	40	160
Confidence Level (95.0%)	0.01^a^	0.06^a^	4.02^b^	2.93^b^	7.01^b^
Two sample *t*-test	<0.0001	<0.0001	<0.0001	<0.0001	

*Superscript “a” was used to indicate absence of risk while superscript “b” was used to indicate the presence of risk in the sampled population*.

## Discussion

Eastern Uganda is an agricultural region of Uganda, predominantly dealing with cattle keeping. Soroti district in particular is one of the largest district in the area with numerous butcheries (points of sale) in the communities. Unfortunately, the beef sold in the district have contaminations including heavy metals, sources of which are anthropogenic in nature. The mean concentrations of heavy metals in the samples, from the highest to the lowest, revealed that Fe, Zn, Cr, Ni, Pb, Co, Cu, and Cd should be of concern in that order. Comparatively, this order is contrary to our earlier finding in the southwestern region of Uganda in which the order was Zn > Pb > Fe > Cu, and no Cd was detected (3). The discrepancy is explained by the fact that these two regions have different anthropogenic activities with potential health risks.

Earlier studies by Kasozi et al. (3) showed that accumulation of heavy metals in beef in both eastern and southwestern regions of Uganda (and by extrapolation, the whole country) is a national crisis requiring close attention by policy making bodies. The National Environmental Authority of Uganda (NEMA), could devise routine monitoring strategies to ensure that the situation doesn't depreciate further. On a broader scale, this falls squarely into the ecological cycle of pollution in Uganda which has been shown to involve everything from waste management (11, 15, 16), sources of natural water (12–14), effect on aquatic wildlife (10), state of drinking water (5, 6) to production of alcoholic beverages (8, 9) as well as food sold to the general public in the streets of Uganda (7). Similar findings of heavy metals have been reported in Kenya fishes and beef (2, 47).

It is well-established that absorption of heavy metals subsequently leads to their bioaccumulation into animal tissues such as beef (50, 51)and into human tissues following consumption of contaminated beef (52–54). This study supports this notion by showing that indeed there were high levels of heavy metals in all samples collected, well above the WHO limits with the exception of Cd. These exceedances of WHO limits point toward carcinogenic and non-carcinogenic risks in the study area (and perhaps a glimpse into what is happening in the entire country, Uganda). In Nigeria similarly, high levels of heavy metals were also found in beef demonstrating the global threat (40). Furthermore, depending on the age group and category of person studied, children, expectant mothers and pastoralists who depend on beef as staple diets in these communities are affected by these contaminants.

The importance of the current study might as well be that of revealing an indicator for heavy metals contamination in the environmental in eastern Uganda [similar to the concept of a bio-indicator species reported in Egypt (55)]. In terms of health risks assessment on the other hand, we show that the estimated daily intake (EDI) for the heavy metals was significantly higher in children than adults. High level of non-essential metals in children is destructive to organs such as kidney and liver during development. Additionally, damage to organs like intestines, reproductive system and skin is not uncommon.

Essential elements were in the order of Fe > Zn > Co > Cu. The order of heavy metals reveals pattern of less amounts of essential nutrients from the beef of eastern Uganda as compared to the recommended daily intake. Daily amounts of 1 ppm/day for Zn, 6 ppm/day for Fe; 56 ppm/day for Co and of 35 ppm/day for Cu required for normal body functioning (18). Since Fe, Cu and Co play a crucial role in erythrocyte physiology and metabolism, there is need for nutritional supplementation in order to curb deficiencies (17, 26).

The estimated daily ingestion of non-essential elements was in the order of Cr > Ni > Pb > Cd and these were generally higher than the tolerable daily intake recommended by WHO except for Cd, hence demonstrating the importance of Pb, Cr and Ni poisoning in eastern Uganda (18, 21, 32, 34). Indeed Pb poisoning was in agreement with our previous study in southwestern Uganda executed by (3).

Overall, our data suggest that there is no health risk associated with consumption of the essential elements Cu, Zn, Fe and Co (THQ < 1). On the other hand, a significantly higher risk of consuming the non-essential elements Cr, Pb, Ni, and Cd in the beef samples studied. The risk was more pertinent in adults than in children, the order of impact of which was Cr > Pb > Ni > Cd. Particularly, Cr, which is associated with urticaria, anemia and generalized visceral disorders (37, 38), appears to be a threat to public health in Soroti. This agrees with epidemiological reports in eastern Uganda where anemia rate was found to be 58.8% and stunted growth to be at 27.7% amongst children (56). Cancer risk was found to be highest in the order of Ni > Cr > Cd > Pb, and significantly higher in children than in adults. Most critical though is that the elements Ni, Cr, Cd, and Pb are all established carcinogens (9, 35, 36) and finding them in beef here at any concentration raises serious concerns because there are no “safe” levels of carcinogens. Furthermore, chronic consumption of Cd in beef would predispose children to cancer in their elderly ages (33).

## Conclusion

In this study, we have shown that toxic heavy metals are present in beef from Soroti (Uganda) at concentrations far higher than those recommended by WHO. We have also shown that essential elements are present at concentrations much lower than those recommended by WHO, hence increasing the risk of deficiency in these necessary enzyme co-factors. The risk of cancer from the consuming Soroti beef was found to be mainly propagated by chronic ingestion of Ni.

Based on the above, we recommend practical livestock production strategies that minimize livestock exposure to heavy metals in eastern Uganda. To effectively do so, we suggest at the minimum a collaborative effort led by the Uganda Ministries of Agriculture Animal Industry & Fisheries, and of Health with the sole purpose of (i) devising practical strategies to improve beef quality and promote healthy beef consumption in the country, and (ii) designing nutritional guidelines to help communities struggling with malnutrition challenge.

## Data Availability Statement

Data files used in the study can be found at https://figshare.com/s/231bb252ceae87d86d12.

## Author Contributions

KK conceptualized the study and drafted the initial manuscript. KK, GZ, and JE designed the study. YH acquired the data. KK, KM, FS, KA, and FA conducted data analysis. AT, FS, RM, FK, TP, HK, RM, PB, HM, PE, LO, JO, LOO, JM, GB, and OO conducted data interpretation. All authors revised it for intellectual content, approved final version to be published and remain in agreement on all aspects of the work.

## Conflict of Interest

The authors declare that the research was conducted in the absence of any commercial or financial relationships that could be construed as a potential conflict of interest.

## Abbreviations

Cd: Cadmium
Co: Cobalt
Cr: Chromium
Cu: Copper
EFSA: European Food Safety Agency
FAO: Food and Agricultural Organization
Fe: Iron
HI: Hazard index
ILCR: Incremental lifetime cancer risk
MAAIF: Ministry of Agriculture Animal Industry and Fisheries
MoH: Ministry of Health
NEMA: National Environmental Management Authority
Ni: Nickel
Pb: Lead
ppm: Parts per million
THQ: Target hazard quotient
US EPA: United States Environment Protection Agency
WHO: World Health Organization
Zn: Zinc.
