# Supplementary figures and images for: Descriptive Analysis of Heavy Metals Content of Beef From Eastern Uganda and Their Safety for Public Consumption
Source: Front Nutr. 2021 Feb 11;8:592340. doi: 10.3389/fnut.2021.592340 (PMC7905049; doi:10.3389/fnut.2021.592340)

## Slide 1
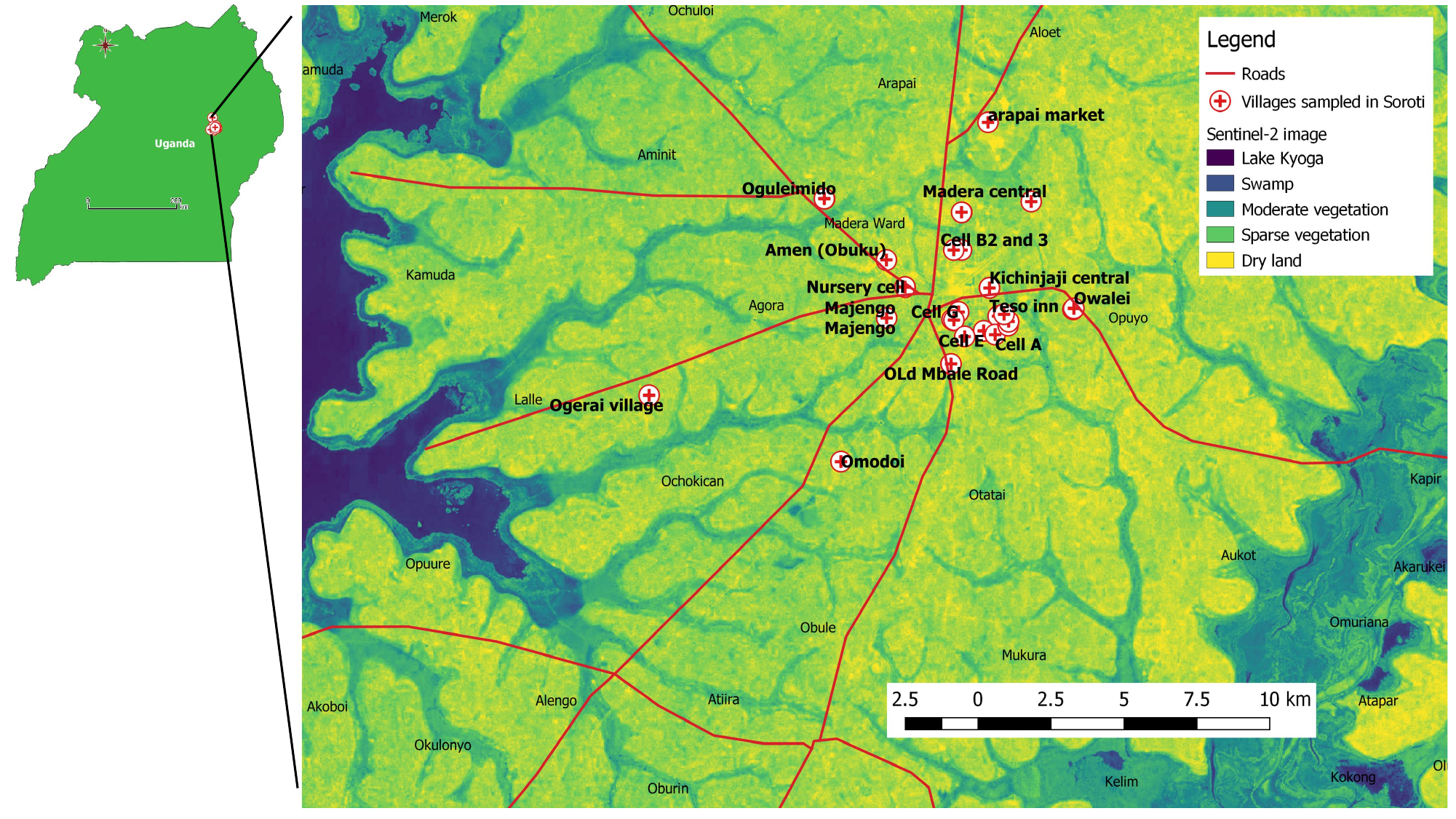

## Slide 2
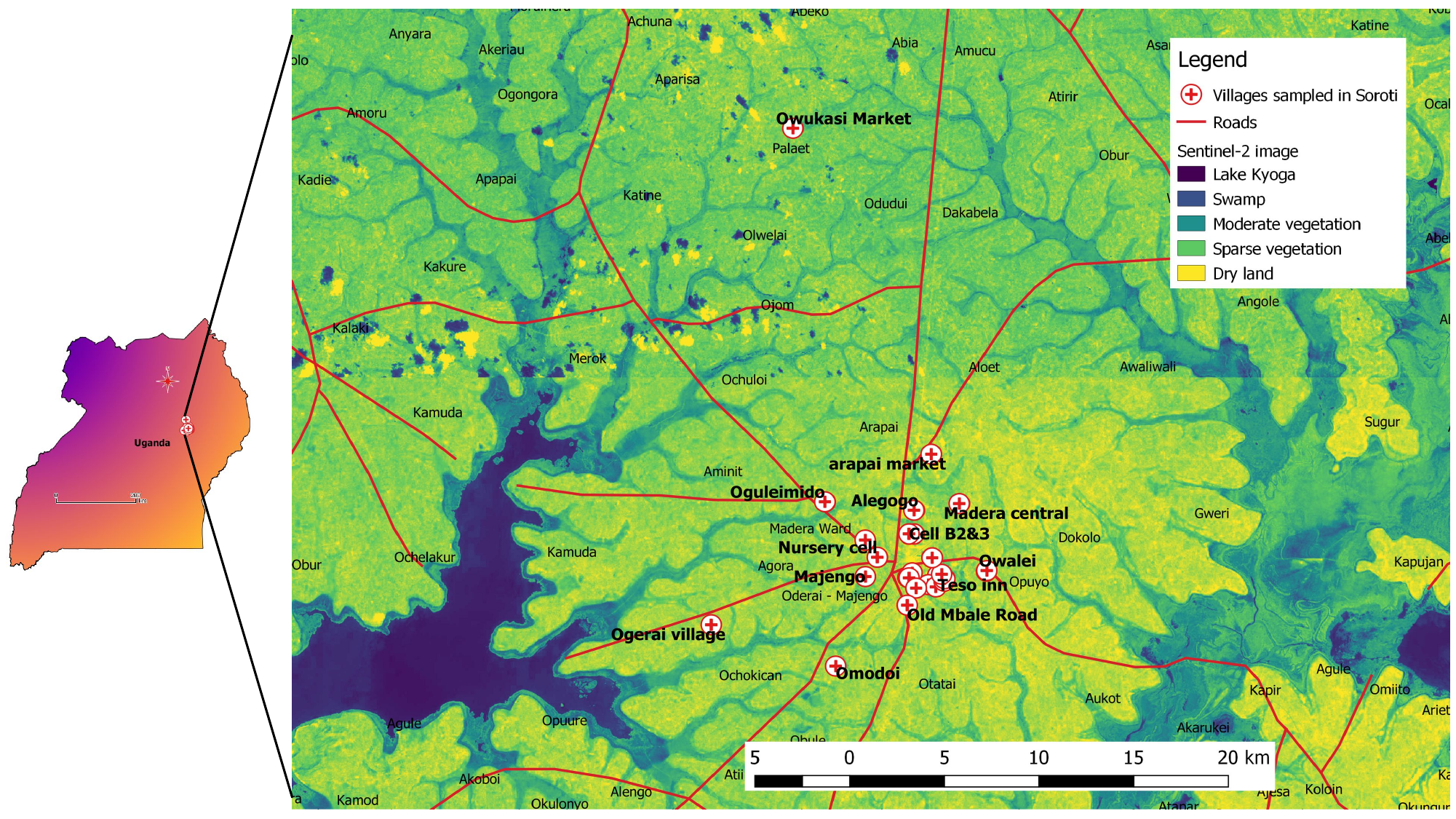

Supplement: Supplementary file 1 [file Presentation_1.PPTX]
